# Prediction of conversion of laparoscopic cholecystectomy to open surgery with artificial neural networks

**DOI:** 10.1186/1471-2482-9-13

**Published:** 2009-08-21

**Authors:** Changiz Gholipour, Mohammad Bassir Abolghasemi Fakhree, Rosita Alizadeh Shalchi, Mehrshad Abbasi

**Affiliations:** 1 Department of General Surgery, Sinaea Hospital, Tabriz University of Medical Sciences Tabriz, Iran; 2 Department of Internal Medicine, Sinaea Hospital, Tabriz University of Medical Sciences Tabriz, Iran; 3 Endocrine and Metabolism Research Center, Vali-asr Hospital, Tehran University of Medical Sciences, Tehran, Iran

## Abstract

**Background:**

The intent of this study was to predict conversion of laparoscopic cholecystectomy (LC) to open surgery employing artificial neural networks (ANN).

**Methods:**

The retrospective data of 793 patients who underwent LC in a teaching university hospital from 1997 to 2004 was collected. We employed linear discrimination analysis and ANN models to examine the predictability of the conversion. The models were validated using prospective data of 100 patients who underwent LC at the same hospital.

**Results:**

The overall conversion rate was 9%. Conversion correlated with experience of surgeons, emergency LC, previous abdominal surgery, fever, leukocytosis, elevated bilirubin and alkaline phosphatase levels, and ultrasonographic detection of common bile duct stones. In the validation group, discriminant analysis formula diagnosed the conversion in 5 cases out of 9 (sensitivity: 56%; specificity: 82%); the ANN model diagnosed 6 cases (sensitivity: 67%; specificity: 99%).

**Conclusion:**

The conversion of LC to open surgery is effectively predictable based on the preoperative health characteristics of patients using ANN.

## Background

Laparoscopic cholecystectomy has proved to be an effective and safe procedure both in elective and emergency conditions; however, conversion to open surgery is inevitable in some cases. The conversion causes elongation of hospital stay, increased total cost, and dissatisfaction of the patients [1]. The common etiologies of such a conversion are uncontrollable bleeding, adhesions, inflammation, anatomical variations, entailed common bile duct (CBD) exploration, trauma of bile duct and other hollow viscera, presence of malignant pathologies, and technical failures. These causal variables are intra-operative events and could not be used as factors to predicate conversions before operations [2,3]. Pre-operative prediction of a laparoscopic cholecystectomy (LC) can assist the surgeon to prepare better for the the risk of conversion to open cholecystectomy[4].

To date, numerous studies have been performed in western countries to predict the conversion of LC to open surgery based on preoperative data [5,6], however, the "artificial neural networks" have not been generally applied. In this study, we analyzed clinical data of 793 patients who underwent elective or emergency LC by linear discrimination, logistic regression, as well as "artificial neural network models" in order to identify risk factors that predict the conversion in East Azerbaijan Province, Iran. We recently published the corresponding data of laparoscopic and open cholecystectomy operations in our center[7].

## Methods

Our study sample comprised patients who underwent LC in the department of surgery at Sinaee Hospital, a teaching university hospital, Tabriz, Iran. A total of 793 consecutive patients (639 females and 154 males) operated between 21^th ^March 1997 and 20^th ^March 2004 were considered for the training group. The preoperative data of patients were extracted from archived data sheets. The data included the following health characteristics and operation conditions: sex, age, history of previous laparatomies, concurrent systemic illnesses (chronic obstructive pulmonary disease, ischemic heart disease, hypertension, chronic renal failure, and diabetes), history of smoking and alcohol use, the surgery setting (emergency or elective) and the surgeon's expertise. Surgeons were considered to be inexperienced in their first 50 LC and experienced afterward. In addition, admission values of body temperature, white blood cell (WBC) count, serum total bilirubin, and serum alkaline phosphatase concentrations, as well as sonographic findings, including gallbladder wall thickness, pericholecystic fluid, CBD stone, and CBD diameter, were collected. The conversion to open surgery and duration of hospital stay was also determined. All the above mentioned data were gathered prospectively for the first 100 LC operations performed since March 2006 in the same department to compose the validation group. The study protocol was approved by the ethics committee of the university and confidential data handling regulations were employed.

### Statistical analyses

For multivariate analysis and to enable prediction of conversion, the forward stepwise logistic regression and the linear discriminant analysis techniques were applied. A probability of 0.05 or less was accepted as statistically significant. The retrospective data of 793 subjects in the training group were used to create a discriminant analysis model. The prospective data of 100 patients of validation group was used to validate the analysis. Two set of regression models were designed to assess the association of predictor variables with conversion. In the first set (bivariate model), each predictor variable was entered into the model and then all the variables were inputted altogether (multivariate). To generate the discrimination function, the following predicting variables were employed: age, sex, history of smoking and alcohol use, presence of concurrent systemic illnesses, history of previous laparatomies, surgeon's experience, emergency/elective setting, laboratory data (bleeding time, WBC count, total bilirubin, and alkaline phosphatase), and sonographic findings including detection of CBD stones, wall thickening, pericystic fluid detection and CBD diameter. The analyses were performed employing SPSS ver. 15 (SPSS Inc.).

The Multi Layered Perceptrons with backpropagation were forward-feed with distinct input, output, and two hidden layers. The errors at the output layer were used to adjust the weights of all the connections immediately preceding the layer in the network using retropropagation of error. The input layer consists of twenty-six nodes, one for each quantitative and more than one for each categorized parameters of the 16 above-mentioned independent variables and 2 neurons (one for each class) in the output layer. The input layer did not contain bias nodes. We selected two hidden layers with 8 and 2 neurons in each hidden layer. The number of nodes in each hidden layer and the number of hidden layers were selected by genetic optimization algorithms with network training for these parameters. The ANN was designed employing NeuroSolutions ver. 5 (NeuroDimention Inc.)

## Results

Table 1 represents the demographic data of training and validation groups. The operations of validation group were performed by more experienced surgeons and were less frequently carried out in emergency conditions (P < 0.001 for both).

**Table 1:** Demographic characteristics and operative conditions of the participants

		Testing set n = 100 male/female:16/84	Training set n = 793 male/female:154/639	Total n = 893
		Mean(SD)	Mean(SD)	Mean(SD)
Age (years)		50.1(15.7)	48.7(14.9)	48.9(15)
Body Temperature (°C)		37(0.3)	36.8(0.7)	36.8(0.7)
WBC count (per mm^3^)		7633.2(2942.4)	7235.1(2195.6)	7279.6(2292.8)
Total bilirubin (mg/dl)		1.2(1.3)	1.2(1.7)	1.2(1.7)
ALK(mg/dl)		260.8(221.6)	233(219.8)	236.1(220.1)
Bleeding time		37.0(0.3)	36.8(0.7)	36.8(0.7)
		Number (%)	Number (%)	Number (%)
Surgeon's Experience (number of LC)	yes	99(99)	507(63.9)^SIG^	606(67.9)
Patient admission type	Emergency	16(16)	680(85.8)^SIG^	696(77.9)
Previous Laparotomy	yes	18(18)	215(27.1)	233(26.1)
Co-Existing Disease	yes	31(31)	193(24.3)	224(25.1)
Smoking	yes	14(14)	66(8.3)	80(9)
Conversion to Open Surgery	yes	9(9)	73(9.2)	82(9.2)

^SIG ^indicates statistically significant differences

**Figure 1:**
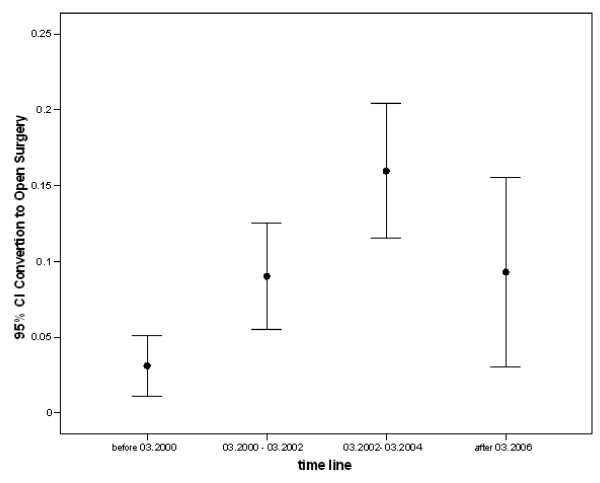
**The trend of conversion of laparoscopic cholecystectomy to open surgeries over the time period of the study**.

In bivariate regression models the training group, conversion of LC to open surgery was associated with inexperience of the surgeon, history of previous laparotomy, history of smoking, higher body temperature, WBC count, alkaline phosphatase, and positive sonographic findings (Table 2). Multivariate analyses showed that among all, experience of surgeon, previous history of laparotomy, CBD stone, body temperature, WBC, bilirubin, and alkaline phosphatase levels were correlated to conversion independent of other variables (Table 2). The prevalence of conversion of LC to open cholecystectomy increased over the training time (Figure 1), whoever, the conversion rate decreased in validation group (2006).

**Table 2:** Factors associated with conversion to open surgery

	Bivariate analysis		Multivariate analysis	
	OR(CI)	P value	OR(CI)	P value
Sex(referent: male sex)	0.65(0.39–1.11)	0.1138	0.81(0.44–1.47)	0.4835
Experience of surgeon	2.71(1.47–4.99)	0.0014	2.28(1.19–4.38)	0.013
Emergency surgery	0.58(0.31–1.09)	0.0922	0.44(0.22–0.88)	0.0202
Previous laparotomy	2.06(1.29–3.29)	0.0026	1.72(1.01–2.93)	0.045
Concurrent disease	1.73(1.07–2.79)	0.0256	1.46(0.85–2.51)	0.1755
Smoking	2.57(1.39–4.75)	0.0026	1.82(0.82–4.05)	0.1412
Drinking	1.68(0.57–4.97)	0.3472	0.75(0.17–3.29)	0.7082
pericholecystic edema	7.66(1.68–34.84)	0.0084	4.94(0.72–33.93)	0.1046
CBD stone	5.26(1.92–14.4)	0.0012	6.91(1.55–30.8)	0.0112
Gallbladder thickening	3.11(1.91–5.06)	0	1.77(0.99–3.15)	0.0526
Age	1.01(1–1.03)	0.0566	1.01(1–1.03)	0.1523
Body Temperature	2.14(1.34–3.41)	0.0015	1.94(1.1–3.41)	0.0217
WBC	1(1-1)	0	1(1–1)	0.0051
bilirubin	1.03(0.92–1.15)	0.6258	0.65(0.43–0.99)	0.0443
alkaline phosphatase	1(1-1)	0.0001	1(1–1)	0.0012
CBD diameter	1.14(1.03–1.26)	0.0087	1(0.87–1.14)	0.9863

OR (CI) represents Odds Ratio and 95% confidence intervals

In the training group, employing linear regression discriminant analyses, sensitivity and specificity of determinants to predict conversion were 51% and 84%; whereas using ANN, these rates rose to 62% and 100%, correspondingly. The models constructed based on the data of validation group – using discrimination analysis – had sensitivity and specificity of 56% and 82%, respectively; the ANN method raised these values to 67% and 99%, correspondingly. The diagnostic quality of these two methods in training and validation groups is compared in table 3.

**Table 3:** Sensitivity, specificity, positive predictive value, and negative predictive value of the training and validation group: comparing linear discriminant analysis and artificial neural network

Group	Statistical accuracy	Discriminant Analysis	artificial neural network
Training	Sensitivity	50.7	61.6
	specificity	84.3	99.4
	positive predictive value	24.7	91.8
	negative predictive value	94.4	96.2

validation	Sensitivity	55.5	66.7
	specificity	82.2	98.9
	positive predictive value	23.8	85.7
	negative predictive value	94.2	96.8

The prediction models were created based on the data of training group.

## Discussion

Our findings revealed that the conversion of LC to open surgery is fairly predictable with preoperative specifications of the patients by ANN. This model was programmed based on the data of 793 LC cases and was validated on another 100 cases. Out of 100 LC procedures in the validation group, 9 cases were complicated and their operations were converted to open surgery. The proposed ANN model could effectively diagnose 6 cases (66%) of conversions before surgery whereas it missed 3 cases, and 1 patient who was successfully operated with laparoscopic approach was falsely classified as a subject whose LC would become complicated with conversion (1%). The traditional discrimination analysis diagnosed 5 out of 9 converted cases at the expense of misclassifying 16 cases as converted group. The prediction however seems to be sensibly acceptable given that more than half of the converted cases were correctly diagnosed.

The discrimination model was not as capable as the ANN model in predicting conversions, however it represents the relevant factors in a more understandable and practical way. The conversion probability could be calculated simply by calculators at patients' bedsides. In contrast, the ANN acts in a way which is called "black box". To apply ANN model, access to the trained software is a necessity compared to discriminant analysis models which simply work with inserting values of the related variables in the formula even at bedside. Nevertheless, the discriminant formula are not trainable while the ANN models can be enhanced according to the information derived from new data and can adopt new conditions, for instance improving surgeons' skills. Furthermore, as the association of predictors may be complicated and demonstrates intricate interrelationships and cross effects, the ANN programming may suit the condition more appropriately and provide more flexible non-linear predictions [8,9]. Although the ANN has shown to be a competent approach in similar situations, [10-16] its practical fitness in clinical conditions still remains to be established.

Based on multivariate regression analyses, we could discuss the predicting factors more understandably [17]. In contrast to numerous previous studies [4,18-21], male sex and age did not influence the conversion. However, there are some reports in line with our finding [22,23]. Adhesions are probably the cause of increasing conversion rate in aged patients [21,24-26]. History of past laparotomy, a predisposing factor for developing adhesions, was found to be an unyielding correlate of conversions in this study in agreement with several previous reports [27,28]. Positive sonographic findings representing higher degrees of inflammation or the necessity for CBD exploration [29-31] were correlated with conversions in bivariate analyses; however, in multivariate analyses, the CBD stone showed to be the single significant determinant. Likewise, fever, leukocytosis, bilirubin, and alkaline phosphatase levels predicted more conversions consistent with previous reports [32,33]. Although past history of concurrent chronic diseases was correlated with conversion, after adjustment for other variables in multivariate model, such history did not predict conversion to open surgery (Table 2). This finding is in agreement [30] and comparable [19,34] with previous studies. Emergent surgery was a risk factor for conversion independent of other variables [35]. Finally and as expected, experience of the surgeon was found to be associated with fewer conversions as it was previously mentioned. The prevalence of conversion of LC to open cholecystectomy increased over the training time and decreased in validation group (Figure 1). This mainly is the result of inclusion of young surgeons into practice experiencing during the training period and less vigilant selection of patient (data not shown).

This study suffers from certain limitations. First of all, the data collection of the training group was performed in a retrospective fashion. This method naturally fails to be as accurate as prospective data collection; however, data of the validation group, which were collected prospectively, corresponded reasonably with that of the training group. Secondly, our study bears the flaws of single centered studies. Third, we regarded a surgeon as experienced after performing 50 LCs. This seems to be a relatively rough criterion to determine the level of experience of a surgeon. Forth, the robustness of the results may be damaged by the differences of characteristics of validation and test groups (i.e. surgeon skill and prevalent emergency surgeries) in particular for discriminant regression analyses. Interestingly, the ANN approach is explicitly appropriate for evaluation of data of shifting populations. Finally, among the parameters that can influence the conversion rate, body mass index was not included in this study.

## Conclusion

The conversion to open surgery is an unyielding complication of laparoscopic cholecystectomy (LC). In this report, employing a relatively large sample size of 893 LC surgeries, we examined predictability of the conversion applying artificial neural network (ANN) models. Our findings suggest that the ANN is superior to traditional discriminant analyses for preoperative prediction of conversion of LC to open surgery.

## Competing interests

The authors declare that they have no competing interests.

## Authors' contributions

CG conceived the study and provided expertise and oversight throughout the process, MBAF designed the ANN, RAS coordinated the study process and data collection, MA drafted the manuscript. All authors participated in data interpretation and statistical analyses, and read and approved the final version.

## Pre-publication history

The pre-publication history for this paper can be accessed here:


